# Comparative evaluation of efficacy, pharmacodynamics, and safety of Hetero’s adalimumab (Mabura®, Hetero Biopharma Ltd.) and reference adalimumab (Humira®, Abbvie Inc.) in patients with active rheumatoid arthritis on concomitant methotrexate therapy

**DOI:** 10.1186/s41927-020-00124-9

**Published:** 2020-06-04

**Authors:** Shubhadeep Sinha, Biswadip Ghosh, Syamasis Bandyopadhyay, Firdaus Fatima, Vamsi Krishna Bandi, Pankaj Thakur, Bala Reddy, Sreenivasa Chary, Leela Talluri, Ajay Gupta, Amit Ramchandra Kale, Anil Kumar Gupta, Ashok Kumar P, Diwakar Reddy, Younus Mohammed, Soma Shekar, Sudheer T, Vijay G. Goni, Vishnu Sharma, Vishwanath Yeligod

**Affiliations:** 1 Hetero Biopharma Limited, Hyderabad, India; 2 Department of Rheumatology, Institute of Post Graduate Medical Education & Research & SSKM Hospital, 244, AJC Bose Road, Kolkata, West Bengal 700020 India; 3 Apollo Gleneagles Hospitals, Kolkata 58, Canal Circular Road, Kolkata, West Bengal 700054 India; 4 Vasavi Medical & Research Centre, 2nd floor, No.6-1-91, Opposite Meera talkies, Khairatabad, Hyderabad, Telangana 500004 India; 5 Opp. MLB Medical College, Nirmal Hospital, Gate no-3, Jhansi, Uttar Pradesh 284128 India; 6 B. J. Medical College, Sassoon General Hospital, Pune, Maharashtra 411011 India; 7 GSVM Medical College, Swaroopa Nagar, Kanpur, U.P 208002 India; 8 Andhra Medical College, Department of Orthopedics, King George Hospital, Visakhapatnam, Andhra Pradesh 530002 India; 9 St.Theresa’s Hospital Erragadda, Sanathnagar, Hyderabad, Andhra Pradesh 500018 India; 10 M.Jeevan Jyoti Multispecialty hospital & Infertility Research Centre, 162 Bai Ka Bagh, Lowther Road, Allahabad, Uttar Pradesh 211003 India; 11 Gurushree Hi-Tech Multi speicality Hopsital No.1558 Opp. Chandra layout Bus Stand Chandra Layout Vijaynagar, Bangalore, Karnataka 560040 India; 12 Department of Orthopedics, Rajiv Gandhi Insititute of Medical Sciences & RIMS Government General Hospital, Srikakulam, 532001 India; 13 Department of orthopedic surgery, Post graduate institute of medical education & Research, Sect, Chandigarh, or-12 India; 14 B.J. Medical College, Civil Hospital, Asarva, Ahmedabad, Gujarat 380016 India; 15 Sapthagiri Institute of Medical Sciences and Research Center #15, Chikkasandra, Hesaraghatta Main Road, Bangalore, Karnataka 560090 India

**Keywords:** Rheumatology, Adalimumab, Methotrexate, Antirheumatic agents

## Abstract

**Background:**

Our study aimed to compare efficacy and safety of Hetero’s adalimumab (Mabura®, Hetero Biopharma Limited) versus reference adalimumab (Humira®, Abbvie Inc.) in Indian patients with active rheumatoid arthritis (RA) concomitant on methotrexate (MTX) therapy.

**Methods:**

Patients (*n* = 168) were randomized (2:1) to receive either test or reference product for 24 weeks with concomitant MTX. Proportion of patients achieving American College of Rheumatology 20 (ACR20) criteria at week 12 was the primary endpoint. Changes in Disease Activity Score of 28 joints–C-reactive protein (DAS28-CRP), Health Assessment Questionnaire–Disability Index (HAQ-DI), and patients achieving ACR20 at week 24, ACR50/70 at weeks 12 and 24 were secondary endpoints.

**Results:**

Patients achieving ACR20 responses with test (96.43%) were similar to reference (96.43%) in intention-to-treat (ITT) analysis at week 12. Proportional difference (PD) between groups (PD [95% CI] 0.0 [− 6.0, 6.0], *p* = 1.000) for ACR20 at week 12 for ITT analysis showed lower limit of the two-sided 95% CI was above the pre-specified noninferiority margin of − 15%. Similar trend in PP analysis (PD [95% CI] 0.0 [− 0.03, 0.07], *p* = 1.000), confirmed therapeutic equivalence. No significant difference was noted between arms for patients attaining ACR20 at week 24 and ACR50/70 at weeks 12 and 24 (all *p* > 0.05). DAS28-CRP and HAQ-DI were similar between groups. Total of 54 patients reported 88 AEs during the study. Out of these, 60 AEs were reported in 34 patients with Hetero-Adalimumab and 28 AEs were reported in 20 patients with Reference-Adalimumab. Total two patients, one in each group reported two serious adverse events (Sinusitis and Viral infection) during the study and resolved completely. No deaths and no life threatening AEs were reported.

**Conclusion:**

Results demonstrated Hetero’s adalimumab is as effective and well tolerated as reference adalimumab in patients with active RA concomitantly on MTX therapy.

**Trial registration:**

CTRI/2016/04/006884, Registered on 28/04/2016.

## Key-points

- Biosimilars may improve patient’s accessibility to biological drug with potentially low prices resulting in reduced treatment cost for patients.
- Hetero Biopharma Ltd. has developed biosimilar adalimumab (Mabura®).
- It is as effective and safe as reference adalimumab (Humira®, Abbvie Inc.) in patients with active rheumatoid arthritis.

## Background

Rheumatoid arthritis (RA), a chronic and progressive autoimmune disease is characterized by persistent inflammation along with erosive joint damage causing functional disability, pain, and premature mortality [1–3]. It occurs in approximately 0.5–1% of the population globally, affecting females more than men (2.5:1.0) [3–6]. At present, there is unavailability of complete cure for RA, and the primary goals of treatment are pain relief, prevention /control of structural damage to the joints, prevention/ reversal of disability and improvement in physical functions and quality of life [7, 8]. The management of RA aims primarily at improving patients’ quality of life (QoL), achieving low disease activity based on American College of Rheumatology (ACR) and European League Against Rheumatism (EULAR) criteria, and ultimately remission. Nonsteroidal anti-inflammatory agents, conventional synthetic disease-modifying antirheumatic drugs (sDMARDs), glucocorticoids and biological disease-modifying antirheumatic drugs (bDMARDs) are the treatment options for RA. Recent treatment advances consider the early use of methotrexate (MTX) with bDMARDs as add-on in patients who are not responding to MTX alone, which has demonstrated improved clinical outcomes and has been approved as the standard of care in patients with moderate-to-severe RA [8–11].

Adalimumab is a recombinant human monoclonal antibody of immunoglobulin G1. It specifically binds to TNF-alpha and inhibits the interaction of tumor necrosis factor (TNF) with surface TNF receptors, thereby reducing clinical symptoms along with ceasing disease progression in patients with RA [12–14]. Adalimumab (Humira®, AbbVie Inc., USA) was first approved in December 2002 by the US Food and Drug Administration (FDA) and is currently approved for multiple immune-mediated inflammatory diseases in addition to RA [15, 16]. Biosimilar development has become imperative to improve patient’s accessibility to bDMARDs with potentially low drug prices and resulting in reduction of treatment cost for healthcare systems and patients [17–19].

The accessibility and affordability of biologic therapies are always a concern in less resourceful regions of the world, leading to limited experience in the clinical use of biologics in such areas. This is of particular concern in countries with developing economies such as India, where treatment costs are mostly borne by the patients, which also impacts the prescription patterns and treatment approaches employed by the physicians. Biosimilars are known to bring down the cost of drug products and thereby increase the access to a larger patient set/population to such therapies, which should improve the sustainability of health care in RA. Mabura (by Hetero Biopharma Ltd., India) is one such adalimumab biosimilar that has been developed for clinical use in India for RA. Recently, numerous biosimilar candidates to Humira® have been developed and studied for comparative efficacy and safety in patients with RA who were on concomitant MTX therapy [20–22]. With this aim, Hetero Biopharma Ltd. has developed a biosimilar adalimumab (Mabura®) for treatment of RA.

As per the World Health Organization (WHO) guidelines, a similar biotherapeutic product (also called a biosimilar) as a “biotherapeutic product that is similar in terms of quality, safety and efficacy to an already licensed reference product”. Mabura (Adalimumab) is a recombinant monoclonal antibody directed to human TNF-α. Mabura® is an IgG antibody composed of two kappa light chains each with a molecular weight of approximately 24 kDa and two IgG1z, a heavy chains each with a molecular weight of approximately 49 kDa. The total molecular weight of Mabura® is 148 kDa. Each light chain consists of 214 amino acid residues and each heavy chain consists of 451 amino acid residues. Hetero has undertaken extensive physical and biological characterisation studies, in vitro, in vivo pre-clinical pharmacological studies with comparability against the innovator (Humira) product. In all these studies, Hetero’s biosimilar Adalimumab was found to be comparable against the innovator reference product. Subsequently, in vivo toxicity studies included single dose acute toxicity and repeated dose toxicity studies of Hetero’s adalimumab versus innovator reference (Humira), which were found to be comparable.

As per the Guidelines of Central Drugs Standard Control Organization, comparative clinical trials are critical to demonstrate the similarity in efficacy and safety profiles between the similar biologic and reference biologic. This study was designed to evaluate and compare efficacy, pharmacodynamics, and safety of subcutaneously administered Hetero’s adalimumab (Mabura®, Hetero Biopharma Limited), referred as “test” in this manuscript, with those of reference adalimumab (Humira®, AbbVie Inc.), referred as “reference” in this manuscript, in Indian patients with active RA who were on concurrent MTX therapy.

## Methods

The study results are presented in accordance with the CONSORT statement.

### Study design

This randomized, prospective, investigator-blinded, multiple-dose, multicenter, comparative, parallel-group study was conducted at rheumatology departments of 15 multi-specialty hospitals/ centers across India from May 2016 to Apr 2017. Patients received 40 mg subcutaneous injection of test or reference product in 2:1 ratio along with MTX (10–25 mg/week) every other week over a period of 24 weeks. Patients who were on stable doses of salicylates, nonsteroidal anti-inflammatory drugs and low doses of corticosteroids (up to 10 mg of prednisolone or equivalent) continued the same dosage till the study completion.

Randomization scheme was generated by permuted block randomization technique by using SAS® (version 9.3 or higher) system software (SAS Institute Inc., USA). Treatment allocation was done centrally after verification of patient eligibility at study sites as per randomization schedule for the study center. This study was an investigator-blinded study to eliminate the assessment bias. Investigators i.e. practicing consulting rheumatologists at the clinical trial sites responsible for the conduct of the clinical trial or designee who assessed study endpoints was blinded to the study medication allocation. Each site has an independent pharmacist who was communicated treatment allocation details and other site team was kept blinded. Independent pharmacist in turn retrieved allocated kit and administered study drug during each visit.

This study was conducted in compliance with the ICH Tripartite guideline regarding Good Clinical Practice and Declaration of Helsinki (Brazil, October 2013) [23], and Schedule Y (amended Drug & Cosmetic Act 2013) [24], and Guidelines for Similar Biologics 2016, India [25] along with subsequent amendments and Indian regulatory laws governing biomedical research in human patients. The study was registered at Clinical Trial Registry-India (CTRI) prior to initiation (CTRI/2016/04/006884) of patient screening. Study was reviewed and approved by institutional ethics committees before its commencement at various sites in India. Written informed consent was obtained from patients before study initiation.

### Participants

Patients of either gender aged ≥18 years to ≤65 years with active RA, concomitantly receiving MTX (10–25 mg/week) for no less than 3 months and on a stable dose between 10 and 25 mg/week for at least 4 weeks were included in this study. Active RA was defined as per the 2010 American College of Rheumatology (ACR)/European League Against Rheumatism (EULAR) classification criteria with RA score of ≥6 and disease duration of at least ≥3 months before baseline. Patients with swollen joints ≥6 (66-joint count), tender/painful joints ≥6 (68-joint count), C-reactive protein (CRP) level of > 6 mg/L and erythrocyte sedimentation rate (ESR) > 28 mm/h [26] were included in the study. Patients with functional class IV as per ACR classification of functional status, receiving DMARDs within 4 weeks before randomization and use of any anti-CD4 therapy, TNF-alpha antagonists, interleukin (IL-1) antagonists, intra-articular/parenteral corticosteroids within 4 weeks prior screening, history of systemic or other chronic infections, systemic manifestations of RA, or those who have used live or attenuated vaccines within 8 weeks before screening were excluded.

### Efficacy and safety assessments

The primary endpoint was to compare the proportion of patients achieving ACR20 criteria at week 12 in between treatment groups. All the patients were evaluated by using ACR response criteria. Patients achieving 20, 50 and 70% improvement in major ACR criteria from baseline to week 12 were considered as ACR20, ACR50 and ACR70 responders. Patients who did not achieve ACR20 at week 12 were classified as treatment failures and withdrawn from the study. Remaining patients continued the treatment up to week 24. Secondary endpoints were proportion of patients achieved ACR20 at week 24, and ACR50 and ACR70 at weeks 12 and 24 in both treatment arms. Patients were assessed for Disease Activity Score 28 joint count–C-reactive protein (DAS28-CRP) and Health Assessment Questionnaire–Disability Index (HAQ-DI) during the study. HAQ (health assessment questionnaire) was administered at baseline, every other week till 8 weeks, 12 weeks (primary analysis), 16 weeks, 20 weeks and at 24 weeks. It included 25 questions across eight categories: Dressing and grooming, Arising, Eating, Walking, Hygiene, Reach, Grip, Common daily activities. IL-6 was assessed as exploratory pharmacodynamic parameter from both treatment arms at baseline and week 12.

Treatment-emergent adverse events (TEAEs) and immunogenicity were assessed as safety endpoints. The immunogenicity assessments were performed for the presence of anti-adalimumab antibodies in all patients of both groups at screening, at the end of week 12 and 24 weeks. The immunogenicity sample analysis was performed by using a validated electrochemiluminescence immunoassay. The sensitivity of this assay was 3.3 ng/ml (USFDA recommends of 250 ng/ml to 500 ng/ml Assay Development and Validation for Immunogenicity Testing of Therapeutic Protein Products- Guidance for Industry- draft guidance- April 2016) with an established drug tolerance up to 20 μg/ml at the low surrogate positive control level. The assay performed in a three-tier strategy with initial screening, confirmatory for screening positives. For clinical safety assessment, patients were monitored for clinical signs and symptoms as well as laboratory abnormalities during treatment.

### Statistical analysis

The sample size was estimated assuming the expected ACR response of 57.2% in test adalimumab and 67.2% in reference adalimumab (based on the Statistical Review of Adalimumab. US FDA) [27]. Non-inferiority margin was selected to preserve at least 50% of the placebo deducted effect size of reference product. Placebo deducted effect size of Abbvie’s Humira was 37% (67.2% in HUMIRA/MTX group compared to 30% in placebo group). Non-inferiority margin of 15% preserves 50% of the placebo deducted effect size of Humira. A sample size of at least 105 subjects were sufficient to prove the non-inferiority of Hetero-Adalimumab compared innovator’s Adalimumab with 80% power and 0.05% of level of significance. However, considering the study drop-outs, and Similar biologics 2016 guidelines of CDSCO, India, 168 patients were randomized with an allocation ratio of 2:1, (112 test-adalimumab arm and 56 reference-adalimumab arm).

Efficacy and safety analysis were performed for intention to treat (ITT) and per protocol (PP) population. The ITT population was defined as patients randomized to receive at least one dose of the study medication at baseline and at least one efficacy evaluation available during evaluation period. The PP population included randomized patients who received study medications and completed all study visits as was defined in the protocol without any major protocol deviations. All patients who received at least one dose of the study drug considered for the safety population. For patients who dropped out of the study for any other reason, the last value was carried forward (LOCF) for primary and secondary analyses. The variables measured on continuous scale such as age, height, the mean, standard deviation, median and range were compared using t-test and the proportions like males/female were compared using Fisher’s exact test. ACR individual criteria (i.e. SJC, TJC, PGA, CRP etc.) were presented as absolute values and presented as point estimates at each visit and compared between groups by using t-test. The change in individual ACR criteria from baseline at each subsequent visit compared within the group by using paired t-test. ACR individual criteria compared between the groups by using Analysis of Covariance (ANCOVA). ACR20, ACR50 and ACR70 responder rates were presented as proportions at each visit and compared between groups by using Fishers exact test. The improvement of DAS28 at each week was summarized by treatment group and compared between groups by using t test. The change form baseline to subsequent visits were compared using ANCOVA. The mean HAQ-DI score (total and each category) was calculated at baseline and subsequent visits. These estimates were compared within and between the treatment groups using t-test. ANCOVA was performed to adjust for the differences in the baseline parameters between the treatment groups. Adverse events (AEs) and adverse drug reactions (ADRs) were summarized by system organ class (SOC) and by preferred terms using the Medical Dictionary for Regulatory Activities Terminology (MedDRA). The incidence of serious adverse events (SAEs), ADRs and AEs were compared across the treatment groups using Fisher’s exact test. All statistical tests were performed using SAS® (version 9.3 or higher) system software (SAS Institute Inc., USA).

## Results

### Patient disposition and characteristics

**Fig. 1:**
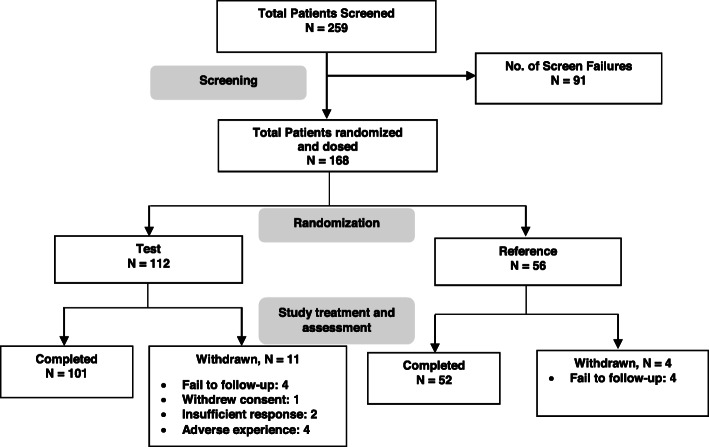
Patient disposition

Overall, 259 patients were screened with active RA at 15 investigational sites across India. Of these, 168 patients who met the eligibility criteria were enrolled and randomized in a 2:1 ratio to receive test (*n* = 112) and reference (*n* = 56) treatments (Fig. 1). In PP analysis, 159 out of 168 patients (107 test adalimumab and 52, reference) who have completed 12 weeks of treatment were included. Overall 153 patients completed 24 weeks of the study period and 15 patients withdrawn from the study. Reasons for withdrawal were adverse experience (*n* = 4), lost to follow-up (*n* = 8), insufficient therapeutic response (*n* = 2), and withdrawal of their consent (*n* = 1) in the study. Demographic characteristics of patients are summarized in Table 1. A majority of patients were female aged 43.90 ± 11.37 years in test and 40.8 ± 9.99 years in reference group. ACR score of patients in test and reference group was 8.9 and 9.08, respectively. Average 66-joint count for swollen joints in patients was 24.3 ± 13.24 and 23.8 ± 11.62 in test and reference groups, respectively. Average 68-joint count for painful/tender joints in patients was 29.1 ± 12.99 in test and 29.9 ± 11.95 in reference group. ACR score, 66-joint count for swollen joints and 68-joint count for painful/tender joints were similar between test and reference groups (Table 1). All other patient characteristics were similar between both treatment groups (Table 1).

**Table 1:** Patients demographic and baseline characteristics

Characteristics	Test (***n*** = 112)	Reference (***n*** = 56)	***p*** value
Gender			
Male	22 (19.6)	9 (16.1)	0.675*
Female	90 (80.4)	47 (83.9)	
Age (years)	43.90 ± 11.37	40.8 ± 9.99	0.088**
Height (cm)	158.27 ± 7.03	156.90 ± 7.22	0.240**
Weight (Kg)	58.58 ± 11.52	56.76 ± 9.47	0.310**
BMI (Kg/m^2^)	23.42 ± 4.88	23.08 ± 3.74	0.646**
Race			
Asian	112 (100)	56 (100)	0.557*
RA Score	8.96	9.08	NA
ACR Response Criteria			
Swollen Joint Count (SJC)	24.3 ± 13.24	23.8 ± 11.62	0.794
Tender Joint Count (TJC)	29.1 ± 12.99	29.9 ± 11.95	0.695
Patient’s Assessment of Pain	80.7 ± 8.77	80.6 ± 10.02	0.957
Patient’s Global Assessment of Disease Activity	79.3 ± 9.90	76.8 ± 11.58	0.162
Physician’s Global Assessment of Disease Activity	76.6 ± 8.85	76.6 ± 9.54	0.995
Patient’s assessment of Physical Function	2.0 ± 0.40	1.9 ± 0.45	0.077
Acute Phase Reactant (CRP (mg/dL))	24.8 ± 28.61	22.7 ± 22.64	0.632
DAS28-CRP	6.6 ± 0.84	6.5 ± 0.83	0.336
HAQ-DI	2.0 ± 0.40	1.9 ± 0.45	0.0775
IL-6 level	15.9 ± 15.78	18.0 ± 20.87	0.705

Data are shown as mean ± SD or n (%); *p values are obtained by performing Fisher’s exact test; **p values are obtained by performing t-test; *DAS28-CRP* Disease activity score in 28 joints C-reactive protein; *HAQ-DI* Health assessment questionnaire disease index, CRP C-reactive protein; *IL-6* Interleukin-6, *TNF-alpha* Tumor necrosis factor-alpha; NA: not applicable

### Efficacy

#### Primary efficacy assessment

ACR20 responses are enlisted in Table 2. In ITT population, ACR20 was achieved in 108 (96.43%) patients with test and 54 (96.43%) patients with reference at week 12. In PP population, ACR20 was achieved in 107 (100%) patients with test and 52 (100%) patients with reference at week 12. For the two-sided 95% CI of the primary endpoint, the lower limits − 6.0 (for ITT) and − 0.03 (for PP) were above the prespecified noninferiority margin of − 15%, showing that test was equally effective as reference in achieving ACR20 in patients having active RA concomitantly on the MTX (10–25 mg/week) therapy (Table 2).

**Table 2:** ACR20, 50, and 70 responses between treatment groups at weeks 12 and 24

	ITT analysis		PP analysis	
	Test (***n*** = 112)	Reference (***n*** = 56)	Test (***n*** = 107)	Reference (***n*** = 52)
**At Week 12**				
ACR20 response (%)	108 (96.43)	54 (96.43)	107 (100)	52 (100)
PD (95% CI)	0.0 (−6.0, 6.0)		0.0 (− 0.03, 0.07)	
*p* value	1.000		1.000	
ACR50 response (%)	27 (24.11)	20 (35.71)	26 (24.30)	19 (36.54)
PD (95% CI)	− 11.6 (− 26.4, 3.2)		−12.2 (− 27.6, 3.2)	
*p* value	0.145		0.134	
ACR70 response (%)	6 (5.36)	6 (10.71)	6 (5.61)	6 (11.54)
PD (95% CI)	−5.4 (−14.5, 3.8)		−5.9 (− 15.6, 3.8)	
*p* value	0.217		0.209	
**At Week 24**				
ACR20 response (%)	104 (92.86)	54 (96.43)	99 (96.12)	51 (100.00)
PD (95% CI)	−3.6 (−10.4, 3.2)		−3.9 (−7.6, 0.2)	
*p* value	0.499		0.303	
ACR50 response (%)	89 (79.46)	44 (78.57)	86 (83.50)	42 (82.35)
PD (95% CI)	0.9 (−12.2, 14.0)		1.1 (−11.5, 13.8)	
*p* value	1.000		1.000	
ACR70 response (%)	54 (48.21)	30 (53.57)	53 (51.46)	29 (56.86)
PD (95% CI)	−5.4 (−21.4, 10.7)		−5.4 (−22.1, 11.3)	
*p* value	0.624		0.608	

*p* values were calculated using Fisher’s exact test; ACR20, ACR50 and ACR70 responses: ≥20%, ≥50%, and ≥ 70%, respectively, improvement in swollen joint count, tender joint count, physician’s assessment of disease activity, patient’s assessment of disease activity, pain, and physical function, and levels of an acute-phase reactant (either C-reactive protein [CRP] level or erythrocyte sedimentation rate [ESR]); *ACR* American College of Rheumatology, *CI* Confidence interval, *ITT* Intention-to-treat, *PP* per-Protocol, *PD* Proportional difference

#### Secondary efficacy assessment

ACR20 achieved in 104 (92.86%) versus 54 (96.43%) patients in ITT analysis and 99 (96.12%) versus 51 (100.00%) patients in PP analysis for test versus reference at week 24, (Table 2). ACR20 response at week 24 was similar between both treatment groups in ITT (*p* = 0.498) and PP (*p* = 0.302) analysis.

In ITT population, ACR50 achieved in 27 (24.11%) & 89 (79.46%) at week 12 & 24 respectively in test group and 20 (35.71%) & 44 (78.57%) at week 12 & 24 respectively in reference group. In PP population, ACR50 achieved in 26 (24.30%) & 86 (83.50%) at week 12 & 24 respectively in test group and 19 (36.54%) & 42 (82.35%) at week 12 & 24 respectively in reference group. ACR50 response at week 12 and 24 was similar between both treatment groups in ITT and PP population. (Table 2). ACR70 achieved in 6 (5.36%) and 54 (48.21%) patients with test and 6 (10.71%) and 30 (53.57%) patients with reference in ITT analysis at week 12 (*p* = 0.217) and week 24 (*p* = 0.623), respectively. Similarly, ACR70 achieved in 6 (5.61%) and 53 (51.46%) patients with test and 6 (11.54%) and 29 (56.86%) patients with reference in PP analysis at week 12 (*p* = 0.209) and week 24 (*p* = 0. 0.608), respectively. (Table 2).

DAS28-CRP improved from baseline to weeks 12 and 24 with a mean change − 2.1 (±1.06) versus − 2.0 (±1.36) and − 3.3 (±1.58) versus − 3.2 (±1.53), respectively, for test versus reference in ITT population and − 2.2 (±1.02) versus − 2.1 (±1.24) and − 3.5 (±1.48) versus − 3.4 (±1.32), respectively, for test versus reference in PP population (Table 3). The improvement in DAS28-CRP was similar between test and reference at weeks 12 (*p* = 0.967 ITT; *p* = 0.945 PP) and 24 (*p* = 0.919 ITT; *p* = 0.997). HAQ-DI improved from baseline to weeks 12 and 24 with a mean change − 1.0 (±0.51) and − 1.3 (±0.54) with test and − 0.9 (±0.50) and − 1.3 (±0.58) with reference in ITT population and − 1.0 (±0.51) and − 1.4 (±0.52) with test and − 1.0 (±0.44) and − 1.3 (±0.50) with reference in PP population. (Table 3). The improvement in HAQ-DI was similar at weeks 12 (*p* = 0.679 ITT; *p* = 0.588 PP) and 24 (*p* = 0.652 ITT; *p* = 0.449 PP). (Table 3).

**Table 3:** DAS28-CRP value and HAQ-DI scores between treatment groups at weeks 12 and 24

	ITT analysis		PP analysis	
	Test (***n*** = 112)	Reference (***n*** = 56)	Test (***n*** = 107)	Reference (***n*** = 52)
**DAS28 CRP value**				
Change from baseline at week 12*	−2.1 (1.06)	− 2.0 (1.36)	−2.2 (1.02)	− 2.1 (1.24)
MD (±SE)	−0.08 (0.19)		−0.04 (0.19)	
95% CI	(−0.5, 0.3)		(− 0.4, 0.3)	
*p* value	0.968		0.945	
Change from baseline at week 24*	−3.3 (1.58)	−3.2 (1.53)	−3.5 (1.48)	−3.4 (1.32)
MD (±SE)	−0.12 (0.26)		−0.09 (0.24)	
95% CI	(−0.6, 0.4)		(−0.6, 0.4)	
*p* value	0.920		0.997	
**HAQ-DI**				
Change from baseline at week 12*	−1.0 (0.51)	−0.9 (0.50)	−1.0 (0.51)	− 1.0 (0.44)
MD (±SE)	−0.02 (0.08)		−0.00 (0.08)	
95% CI	(−0.2, 0.1)		(−0.2, 0.2)	
*p* value	0.679		0.588	
Change from baseline at week 24*	−1.3 (0.54)	−1.3 (0.58)	− 1.4 (0.52)	−1.3 (0.50)
MD (±SE)	−0.06 (0.09)		−0.03 (0.09)	
95% CI	(−0.2, 0.1)		(−0.2, 0.1)	
*p* value	0.653		0.449	

*Data presented as mean ± SD; p* values were obtained using paired t-test; *p < 0.001 vs. baseline; *CI* Confidence interval, *DAS28-CRP* Disease Activity Score 28–C-Reactive Protein, *HAQ-DI* Health Assessment Questionnaire–Disability Index, *ITT* Intention-to-treat, *PP* Per-protocol, *MD* Mean difference, *SE* Standard error

Change in IL-6 from baseline to week 12 is enlisted in Table 4. The difference in IL-6 mean between test and reference was similar in ITT population (2.60 [− 9.2, 14.4], *p* = 0.878) and PP population (5.30 [− 7.3, 17.9], *p* = 0.436).

**Table 4:** Comparison of exploratory pharmacodynamic parameter between treatment groups at week 12

	ITT analysis		PP analysis	
	Test (***n*** = 27)	Reference (***n*** = 13)	Test (***n*** = 27)	Reference (***n*** = 11)
**Interleukin-6**				
Change from baseline at week 12	−8.2 (15.97)*	−10.8 (19.86)^#^	−8.2 (15.97)*	−13.5 (20.44)^$^
MD (±SE)	2.60 (5.84)		5.30 (6.20)	
95% CI	(−9.2,14.4)		(−7.3, 17.9)	
*p* value	0.878		0.436	

*p* values were obtained using paired t-test; *p = 0.012 vs. baseline; #*p* = 0.0727 vs. baseline; $*p* = 0.0525 vs. baseline

*CI* Confidence interval, *ITT* Intention-to-treat, *PP*, Per protocol, *MD* Mean difference, *SE* Standard error

### Safety

During the study period, 54 patients reported 88 adverse events (AEs). Among them, 34 (30.4%) patients from the test group reported 60 (53.6%) AEs, while 20 (35.7%) patients from the reference group reported 28 (50%) AEs (Table 5). Two patients (one from each group) reported two serious adverse events (SAEs) (sinusitis and viral infection) during the study. Both SAEs were considered related to the study drugs and resolved completely. No deaths or life-threatening AEs were reported in either treatment group. All reported AEs resolved completely without any consequence. Immunogenicity evaluations showed that overall, 53 (61.63%) and 51 (61.45%) patients with test, and 23 (60.53%) and 24 (63.16%) patients with reference treatment developed ADAs at weeks 12 and 24, respectively (Table 5). Similar incidence of ADAs at weeks 12 and 24 were reported in both the treatment groups (*p* = 1.000). During the study, no clinical and physical signs related to safety were observed in either treatment group.

**Table 5:** Incidence of ADAs at weeks 12 and 24, AEs, and TEAEs between treatment groups

	Test (***n*** = 112)	Reference (***n*** = 56)
**Immunogenicity assessment**		
Incidence of ADAs at week 12, n (%)	53 (61.63)	23 (60.53)
PD (95% CI)	1.1 (−17.5, 19.7)	
*p* value	1.000	
Incidence of ADAs at week 24, n (%)	51 (61.45)	24 (63.16)
PD (95% CI)	−1.7 (−20.3, 16.9)	
*p* value	1.000	
**Safety assessments**		
Patients with at least one AE, n (%)	34 (30.4)	20 (35.7)
*p* value	0.4889	
Number of TEAEs, n (%)	60 (53.6)	28 (50.0)
*p* value	0.7436	

*p* values were obtained using paired t-test; *ADAs* Antidrug antibodies, *AE* Adverse event, *TEAE* Treatment-emergent adverse event, *PD* Proportional difference, *CI* Confidence interval

## Discussion

In this prospective, randomized, investigator-blinded, multiple-dose, multicenter, comparative, parallel-group study, safety and efficacy of test were compared with those of reference in Indian patients with active RA concomitant on MTX therapy, in terms of improvement in ACR 20, 50, 70, DAS 28 – CRP scores and HAQ-DI for efficacy assessments, treatment emergent immunogenicity and AEs for safety assessments over the period of treatment.

As USFDA recommends ACR20, a preferred parameter to assess efficacy of new drugs for RA with respect to the signs and symptoms of disease, ACR20 was considered as the primary efficacy endpoint in our study [28] Regulatory authorities suggest week 12 as a sensitive time point for assessing the rapidity of responses in biosimilar comparability studies on RA [28, 29]. Therefore, for ACR20, week 12 was considered as the beginning of the time response curve plateau. In the present study, our primary efficacy endpoint, i.e., response to ACR20 was achieved at week 12 demonstrating non inferiority of test compared with reference. In addition, sensitivity analyses of the primary endpoint with the PP population aided the conclusion of therapeutic equivalence. Previously, Fleischman et al. compared PF-06410293, adalimumab biosimilar, versus Humira® and reported 68.7% versus 72.7% patients who achieved ACR20 at week 12 [29]. Prasad Apsangikar et al. reported ACR20 response at week 16 was 90.48% in study arm and 90% in the reference arm. The number of patients ACR70 response at week 16 was 13.1% in the study arm and 15% in the reference arm (*P* > 0.05) [30]. The primary efficacy results of our study are in agreement with results reported in previous studies for treatment of patients with active RA who were concomitantly taking MTX [20, 22, 29, 30]. Moreover, results of ACR core criteria demonstrated that the efficacy of test is non-inferior to that of reference for achieving ACR20/50/70 at weeks 12 and 24.

In this study, DAS28-CRP was selected with a cut-off of < 2.6 as “remission” and ≤ 3.2 as “low disease activity” [31]. Change in DAS28-CRP was observed similar between test and reference, thus supporting therapeutic equivalence. During the study period, HAQ-DI decreased with no significant difference between both treatment arms. Results for HAQ-DI were in agreement with previous studies that demonstrated improvement in social and physical functions in patients with active RA [21, 32, 33]. Assessment of exploratory pharmacodynamic parameter showed similar reduction in IL-6, which supports similar efficacy of test and reference.

Immunogenicity is the ability of an antigen (in this study, test or reference adalimumab) to elicit an immune response, resulting in ADA formation. ADAs can be either neutralizing or non-neutralizing in nature. Neutralizing antibodies bind to the receptor site and neutralize it, thus possibly prevent or reduce the ability of treatments [34, 35]. The immunogenicity profile of test product was similar to that of reference product during the study period; however, the incidence for ADAs was slightly lower with test product at week 24. Immunogenicity of test and reference showed that positive binding antibodies had not affected ACR20 response in patients. These results were also consistent with previously reported immunogenicity assessment of adalimumab [22]. Comparable safety profiles demonstrated that treatment with test was safe and well tolerated as treatment with reference.

The limitations of the study include the unavailability of older and juvenile RA patients who could benefit from the drug and couldn’t be included because of restrictions in admission criteria. Also, the study data is limited to Indian patients. Safety in other racial populations may need testing, however, the promising efficacy and safety data in this study populations could also mean likelihood of efficacy in RA in other racial populations.

## Conclusion

Results of this study demonstrated no clinically meaningful difference in efficacy, pharmacodynamics, and safety between test and reference treatment groups. Hence, the test adalimumab is being equally efficacious and safe biosimilar to the reference adalimumab for treatment of active RA in patients concomitantly on MTX therapy.

## Data Availability

Data supporting the findings are presented within the manuscript and additional datasets used and/or analysed during the current study are available from the corresponding author on reasonable request.

## Abbreviations

ACR: American college of rheumatology
ACR20: American college of rheumatology 20% improvement
ADA: Antidrug antibodies
AEs: Adverse events
ANCOVA: Analysis of covariance
bDMARDs: Biologic disease-modifying antirheumatic drugs
CI: Confidence interval
CRP: C-reactive protein factor
DAS28-CRP: Disease activity score 28 joint count–C-reactive protein
ELISA: Enzyme linked immunosorbent assay
ESR: Erythrocyte sedimentation rate
EULAR: European league against rheumatism
FDA: Food and drug administration
HAQ-DI: Health assessment questionnaire–disability index
IL: Interleukin
ITT: Intent to treat
LOCF: Last value carried forward
MTX: Methotrexate
PGA: Patient’s global assessment
PP: Per protocol
RA: Rheumatoid arthritis
SAE: Serious adverse events
SC: Subcutaneous
SD: Standard deviation
SJC: Swollen joint count
sDMARDs: Synthetic disease-modifying antirheumatic drugs
TJC: Tender joint count
TEAEs: Treatment-emergent adverse events
TNF: Tumor necrosis factor

## References

[1] Hazes JM (2003). Determinants of physical function in rheumatoid arthritis: association with the disease process. Rheumatology.

[2] Wasserman AM (2011). Diagnosis and management of rheumatoid arthritis. Am Fam Physician.

[3] Kvien TK (2004). Epidemiology and burden of illness of rheumatoid arthritis. Pharmacoeconomics.

[4] Laiho K, Tuomilehto J, Tilvis R (2001). Prevalence of rheumatoid arthritis and musculoskeletal diseases in the elderly population. Rheumatol Int.

[5] Sangha O (2000). Epidemiology of rheumatoid arthritis. Rheumatology.

[6] Crowson CS, Matteson EL, Myasoedova E (2011). The lifetime risk of adult-onset rheumatoid arthritis and other inflammatory autoimmune rheumatic diseases. Arthritis Rheum.

[7] Felson DT, Smolen JS, Wells G (2011). American College of Rheumatology/European league against rheumatism provisional definition of remission in rheumatoid arthritis for clinical trials. Arthritis Rheum.

[8] Aletaha D, Smolen JS (2018). Diagnosis and Management of Rheumatoid Arthritis. Jama..

[9] Mota LM, Cruz BA, Brenol CV (2012). Brazilian Society of Rheumatology Consensus for the treatment of rheumatoid arthritis. Rev Bras Reumatol.

[10] Smolen JS, Landewé R, Breedveld FC (2010). EULAR recommendations for the management of rheumatoid arthritis with synthetic and biological disease-modifying antirheumatic drugs. Ann Rheum Dis.

[11] Singh JA, Furst DE, Bharat A (2012). 2012 update of the 2008 American College of Rheumatology recommendations for the use of disease-modifying antirheumatic drugs and biologic agents in the treatment of rheumatoid arthritis. Arthritis Care Res.

[12] Weinblatt ME, Keystone EC, Furst DE (2003). Adalimumab, a fully human anti–tumor necrosis factor α monoclonal antibody, for the treatment of rheumatoid arthritis in patients taking concomitant methotrexate: the ARMADA trial. Arthritis Rheum.

[13] Keystone EC, Kavanaugh AF, Sharp JT (2004). Radiographic, clinical, and functional outcomes of treatment with adalimumab (a human anti–tumor necrosis factor monoclonal antibody) in patients with active rheumatoid arthritis receiving concomitant methotrexate therapy: a randomized, placebo-controlled, 52-week trial. Arthritis Rheum.

[14] Breedveld FC, Weisman MH, Kavanaugh AF (2006). The PREMIER study: a multicenter, randomized, double-blind clinical trial of combination therapy with adalimumab plus methotrexate versus methotrexate alone or adalimumab alone in patients with early, aggressive rheumatoid arthritis who had not had previous methotrexate treatment. Arthritis Rheum.

[15] US Food and Drug Administration, Guidance for industry: scientific considerations in demonstrating biosimilarity to a reference product. Rockville, Md., US Food and Drug Administration. 2015. .https://www.fda.gov/downloads/drugs/guidances/ucm291128.pdf. Accessed 18 Apr 2019.

[16] Castañeda-Hernández G, González-Ramírez R (2015). Biosimilars in rheumatology: what the clinician should know. RMD Open.

[17] Mulcahy A, Predmore Z, Soeren M. The cost savings potential of biosimilar drugs in the United States. 2014. https://www.rand.org/content/dam/rand/pubs/perspectives/PE100/PE127/RAND_PE127.pdf. Accessed 18 Apr 2019.

[18] Crespi-Lofton J, Skelton JB (2017). The growing role of biologics and biosimilars in the United States: perspectives from the APhA biologics and Biosimilars stakeholder conference. J Am Pharm Assoc.

[19] Meher BR, Balan S, Mohanty RR (2019). Biosimilars in India; current status and future perspectives. J Pharm Bioallied Sci.

[20] Cohen SB, Alonso-Ruiz A, Klimiuk PA (2018). Similar efficacy, safety and immunogenicity of adalimumab biosimilar BI 695501 and Humira reference product in patients with moderately to severely active rheumatoid arthritis: results from the phase III randomised VOLTAIRE-RA equivalence study. Ann Rheum Dis.

[21] Jamshidi A, Gharibdoost F, Vojdanian M (2017). A phase III, randomized, two-armed, double-blind, parallel, active controlled, and non-inferiority clinical trial to compare efficacy and safety of biosimilar adalimumab (CinnoRA®) to the reference product (Humira®) in patients with active rheumatoid arthritis. Arthritis Res Ther..

[22] Fleischmann RM, Alten R, Pileckyte M (2018). A comparative clinical study of PF-06410293, a candidate adalimumab biosimilar, and adalimumab reference product (Humira®) in the treatment of active rheumatoid arthritis. Arthritis Res Ther..

[23] WMA Declaration of Helsinki – Ethical Principles for Medical Research Involving Human Subjects. https://www.wma.net/policies-post/wma-declaration-of-helsinki-ethical-principles-for-medical-research-involving-human-subjects/ Accessed on 10^th^ January 2020..

[24] “SCHEDULE Y s [See rules 122A, 122B, 122D, 122DA, 122DAA and 122E] REQUIREMENTS AND GUIDELINES FOR PERMISSION TO IMPORT AND / OR MANUFACTURE OF NEW DRUGS FOR SALE OR TO UNDERTAKE CLINICAL TRIALS. Reference: https://rgcb.res.in/documents/Schedule-Y.pdf Accessed on 10^th^ January 2020.

[25] GUIDELINES ON SIMILAR BIOLOGICS: Regulatory Requirements for Marketing Authorization in India, 2016.Reference: http://nib.gov.in/NIB-DBT2016.pdf Accessed on 10^th^ January 2020. 10.5731/pdajpst.2012.00886 23035022

[26] Aletaha D, Neogi T, Silman AJ, et al. 2010 rheumatoid arthritis classification criteria: an American College of Rheumatology/European league against rheumatism collaborative initiative. Arthritis Rheum. 2010;62(9):2569–81. 10.1002/art.27584. 10.1002/art.27584 20872595

[27] Statistical Review of Adalimumab. US FDA https://www.accessdata.fda.gov/drugsatfda_docs/nda/2017/761058Orig1s000StatR.pdf Accessed on 10^th^ January 2020.

[28] Felson, LaValley (2014). The ACR20 and defining a threshold for response in rheumatic diseases: too much of a good thing. Arthritis Res Ther.

[29] Jani RH, Gupta R, Bhatia G (2016). A prospective, randomized, double-blind, multicentre, parallel-group, active controlled study to compare efficacy and safety of biosimilar adalimumab (Exemptia; ZRC-3197) and adalimumab (Humira) in patients with rheumatoid arthritis. Int J Rheum Dis.

[30] Apsangikar P, Chaudhry S, Naik M (2018). A prospective, randomized, double- blind, comparative clinical study of efficacy and safety of a biosimilar Adalimumab with innovator product in patients with active rheumatoid arthritis on a stable dose of methotrexate. Indian J Rheumatol.

[31] Fleischmann R, van der Heijde D, Koenig AS (2015). How much does disease activity score in 28 joints ESR and CRP calculations underestimate disease activity compared with the simplified disease activity index?. Ann Rheum Dis.

[32] Burmester GR, Matucci-Cerinic M, Mariette X (2014). Safety and effectiveness of adalimumab in patients with rheumatoid arthritis over 5 years of therapy in a phase 3b and subsequent postmarketing observational study. Arthritis Res Ther.

[33] Burmester GR, Mariette X, Montecucco C (2007). Research in active rheumatoid arthritis trial study group: Adalimumab alone and in combination with disease-modifying drugs for the treatment of rheumatoid arthritis in clinical practice: the research in active rheumatoid arthritis (ReAct) trial. Ann Rheum Dis.

[34] Bartelds GM, Krieckaert CLM, Nurmohamed MT (2011). Development of antidrug antibodies against adalimumab and association with disease activity and treatment failure during long-term follow-up. JAMA..

[35] Bartelds GM, Wijbrandts CA, Nurmohamed MT (2007). Clinical response to adalimumab: relationship to anti-adalimumab antibodies and serum adalimumab concentrations in rheumatoid arthritis. Ann Rheum Dis.

